# Bacterial coinfections and secondary infections in COVID-19 patients from a tertiary care hospital of northern India: Time to adhere to culture-based practices

**DOI:** 10.5339/qmj.2021.62

**Published:** 2021-10-25

**Authors:** Bhawna Sharma, Priya Sreenivasan, Manisha Biswal, Varun Mahajan, Vikas Suri, Inderpaul Singh Sehgal, Pallab Ray, Goverdhan Dutt Puri, Ashish Bhalla, Lakshmi Narayana Yaddanapudi, Vipin Koushal, Archana Angrup

**Affiliations:** ^1^Department of Medical Microbiology, Postgraduate Institute of Medical Education and Research, Chandigarh, India. E-mail: archanaangrup@yahoo.com; ^2^Department of Anaesthesia, Postgraduate Institute of Medical Education and Research, Chandigarh, India.; ^3^Department of Internal Medicine, Postgraduate Institute of Medical Education and Research, Chandigarh, India.; ^4^Department of Pulmonary Medicine, Postgraduate Institute of Medical Education and Research, Chandigarh, India.; ^5^Department of Hospital Administration, Postgraduate Institute of Medical Education and Research, Chandigarh, India.

**Keywords:** COVID-19, secondary bacterial infections, bacterial co-infection

## Abstract

Objective: Bacterial co-pathogens are common in various viral respiratory tract infections, leading to increased disease severity and mortality. Still, they are understudied during large outbreaks and pandemics. This study was conducted to highlight the overall burden of these infections in COVID-19 patients admitted to our tertiary care hospital, along with their antibiotic susceptibility patterns.

Material and methods: During the six-month study period, clinical samples (blood samples, respiratory samples, and sterile body fluids, including cerebrospinal fluid [CSF]) of COVID-19 patients with suspected bacterial coinfections (at presentation) or secondary infections (after 48 hours of hospitalization) were received and processed for the same.

Results: Clinical samples of 814 COVID-19 patients were received for bacterial culture and susceptibility. Out of the total patient sample, 75% had already received empirical antibiotics before the samples were sent for analysis. Overall, 17.9% of cultures were positive for bacterial infections. Out of the total patients with bacterial infection, 74% (108/146) of patients had secondary bacterial infections (after 48 hours of hospitalization) and 26% (38/146) had bacterial coinfections (at the time of admission). Out of the 143 total isolates obtained, the majority (86%) were gram-negative organisms, of which *Acinetobacter species* was the commonest organism (35.6%), followed by *Klebsiella pneumoniae* (18.1%). The majority (50.7%) of the pathogenic organisms reported were multidrug resistant.

Conclusion: The overall rate of secondary bacterial infections (SBIs) in our study was lower (7.9%) than reported by other studies. A rational approach would be to adhere to the practice of initiating culture-based guidance for antibiotics and to restrict unnecessary empirical antimicrobial therapy.

## Introduction

The coronavirus disease 2019 (COVID-19), first identified in December 2019 in Wuhan, China, is a highly pathogenic and transmissible infection caused by severe acute respiratory syndrome coronavirus 2 (SARS-CoV-2).^1^ India has reported 31,257,720 COVID-19 cases as of 22 July, 2021.^2^ Bacterial co-pathogens are common in viral respiratory tract infections, leading to increased disease severity and mortality. Prolonged hospitalization due to respiratory viral infections also predisposes patients to hospital-acquired infections/secondary bacterial infections (SBIs). Despite the proven importance of secondary bacterial infections (SBIs) affecting the severity of viral respiratory diseases, they are still understudied during large outbreaks of viral respiratory infections.^3,4^ There remains a knowledge gap in the nature, frequency, and antimicrobial profiles of secondary bacterial pathogens in the current COVID-19 pandemic.^5,6^ Due to this knowledge gap and paucity of literature, the majority of patients tend to receive unnecessary empirical antibiotics, with no adherence to the antimicrobial stewardship guidelines. According to current reports, almost half of COVID-19 deaths are associated with SBIs or coinfections.^7^ Extrapolating from the concerns of increased mortality seen due to bacterial superinfections during previous influenza pandemics, various guidelines on the empirical use of antibiotics in COVID-19 patients have been advocated.^8–10^ Moreover, with the current scenario, the problem of increasing antimicrobial resistance will likely outlive COVID-19, and hence unnecessary use of antibiotics in the treatment of this pandemic virus should be reduced. A growing number of reports have suggested that antimicrobial stewardship has suffered and that even fundamental principles have been overlooked during the pandemic.^11^

Clinical indications and microbiological evidence should always be kept in mind while treating SBIs in COVID-19 patients. It has been observed that even before the microbiological confirmation of SBIs, the clear majority of COVID-19 patients were given empirical antimicrobial treatment.^9^ Hence, in this current pandemic era, there is increased overuse of empirical antibiotics without their actual need, and this practice will certainly lead to the emergence of antimicrobial resistance in times to come. The increased antimicrobial resistance will pose a real threat to the nation by limiting treatment options, and higher class of antibiotics such as colistin and tigecycline will remain the treatment of choice in that scenario. These higher classes of antibiotics will further increase the rate of patient mortality due to their poor outcomes. Antimicrobial stewardship programs should therefore focus on adherence to the practice of initiating culture-based guidance for antimicrobial therapy and restricting the use of unnecessary empirical antimicrobial therapy. With this background of bacterial coinfections or secondary infections in COVID-19 patients, our study highlighted the overall burden of these infections in COVID 19 patients admitted to our tertiary care hospital.

## Material And Methods

### Study setting and design

This was a prospective, observational study conducted over six months (from May 1, 2020 to October 31, 2020) at the Postgraduate Institute of Medical Education and Research (PGIMER), Chandigarh, a tertiary care hospital in northern India. PGIMER is a 2300-bed tertiary care hospital that caters to a population of approximately 367 million people, primarily from the northwest Indian states, and includes referrals from the rest of the country.

During this study period, clinical samples (blood samples, respiratory samples including sputum and tracheal aspirates, and sterile body fluids including CSF) of COVID-19 patients confirmed by COVID-19 RT-PCR admitted at the dedicated COVID hospital in our institute with a clinical suspicion of SBIs (after 48 hours of hospitalization) or coinfections (at the time of admission) were received in the microbiology laboratory. The clinical and outcome data were obtained from the patients’ medical records.

### Patient enrolment

All patients diagnosed with COVID-19 by reverse transcriptase real-time PCR and with a clinical suspicion of secondary bacterial infections or coinfections (after 48 hours of hospitalization or at the time of admission) were included in the study.

### Sample collection, processing, and identification

A total of 814 clinical samples (645 blood; 124 respiratory [104 tracheal aspirates and 20 sputum samples]; 19 sterile body fluids [6 ascitic fluids, 10 pleural fluids, 2 intraocular fluids, and 1 peritoneal dialysis fluid]; and 26 CSF) were collected using the recommended personal protective equipment guidelines. Repeat samples of the same patients were excluded from the study if similar results were obtained. Identification was performed by matrix-assisted laser desorption ionization-time of flight spectrometry (Vitek - bioMérieux) and was followed by antibiotic susceptibility testing. Respiratory samples were inoculated on blood agar and MacConkey agar and incubated overnight at 37°C. For sterile body fluids, blood and MacConkey agar along with Robertson's cooked meat broth were incubated for 48 hours. An additional chocolate agar was used for CSF samples. Samples for blood culture were received in BACTEC® bottles and were incubated in a BACTEC® 9240 system (Becton Dickinson, Heidelberg, Germany). Positive samples were further processed for identification and antimicrobial susceptibility testing.

### Antimicrobial susceptibility testing

Antimicrobial susceptibility testing (AST) of the clinical isolates was performed using the gram-negative and gram-positive Vitek2® AST cards (N280, N281, and P628) (bioMérieux, Inc., Durham, NC), as per the manufacturer's instructions. The minimum inhibitory concentration for colistin was determined by the Clinical and Laboratory Standards Institute (CLSI) broth microdilution method.^12^ The antibacterial drugs tested against gram-negative pathogens included amikacin, cefepime, cefoperazone/sulbactam, ceftazidime, ciprofloxacin, levofloxacin, imipenem, meropenem, piperacillin/tazobactam, and chloramphenicol. The antibacterial drugs tested against gram-positive organisms included vancomycin, teicoplanin, ciprofloxacin, linezolid, erythromycin, clindamycin, and doxycycline. Multidrug resistance was defined as resistance to two or more different classes of antimicrobials.^13^ After processing, the samples were discarded as per the COVID-19 biomedical waste management guidelines.^14^

### Statistical analysis

Categorical variables such as the number of patients and resistant organisms were expressed as a number, n (%). Age was presented as mean ± standard deviation (SD). A *p-*value < 0.05 was considered to indicate statistical significance. Data were analyzed using Microsoft Excel version 16 (Microsoft Corp., Richmond, CA, USA), and statistical analysis was performed using GraphPad Prism V.6.0 (GraphPad Software, La Jolla, CA).

### Ethical approval

The study was approved by the Institute Ethics Committee with reference no. NK/6623/Study/057.

## Results

### Demographic details

A total of 1844 COVID-19-positive patients were admitted during the study period. Blood (645), respiratory (124), and sterile body fluid (19) samples of 814 patients were received for bacterial culture and susceptibility tests. Out of the total patient sample, 75% patients had already received empirical antibiotics before the samples were sent. Overall, 146 (17.9%) cultures were positive and were included for demographic, severity of illness (based on intensive care unit [ICU] admissions), and outcome measures analyses (Table 1). Among these patients, 58 (39.7%) were of ≥ 55 years and 88 (60.3%) were < 55 years of age. The incidence of secondary infections in the ≥ 55-year age group was higher than that in the < 55-year age group (26% vs 16.1%). Out of 146 culture-positive samples, 53.4% had positive blood cultures, 42.5% had positive respiratory cultures, and sterile body fluid cultures were positive in 4.1% patients. 78 (12.1%) of the 645 total blood samples received, 62 (50%) of the 124 respiratory samples, and 6 of the 19 body fluids (4 pleural fluid, 1 ascitic fluid, and 1 intraocular fluid) were positive for bacterial infections. The 26 CSF samples received were negative for bacterial infections.

### Severity of illness and outcome measures

The severity of illness and outcome measures were based on admission to ICU and in-hospital mortality, respectively. Out of 1844 total COVID-19 admissions during the study period, 373 (20.2%) required ICU admission, and out of these 373 patients, 94 (25.2%) were positive for bacterial infections. Overall mortality was 17% among 1844 COVID-19 patients and 26.8% among 373 ICU patients. However, out of a total of 146 patients whose samples were positive for SBIs, 78 (53.4%) succumbed to the disease due to COVID-19-induced acute respiratory distress syndrome (ARDS) along with septic shock. The mortality rate among patients without bacterial infection (668) was 24%, and the difference between mortality in patients with and without bacterial infections was significant (p < 0.00001) (Table 2). Most of the patients, 62.8% (49/78), who succumbed to COVID-19 were admitted to the ICU, while non-ICU in-hospital mortality remained lower at 55.8% (29/52). There was no statistical difference between the average age (mean ± SD) of patients who had a fatal outcome (48 ± 17.43 years) and those who survived (48 ± 17.41 years) (p>0.05).

### Etiological profile of secondary bacterial infections/coinfections

**Figure 1.**
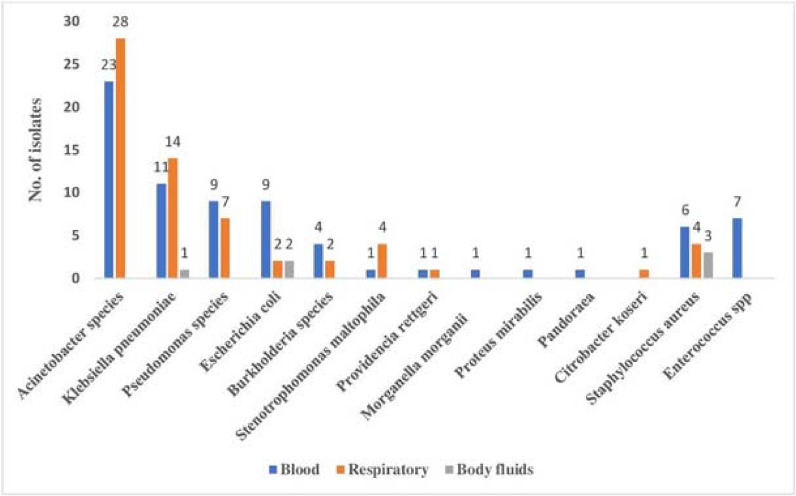
Etiological distribution of bacterial infections in patients hospitalized with COVID-19 (n = 146) among different clinical samples (blood, n = 78; respiratory, n = 62; and sterile body fluids, n = 6).

A total of 814 samples (blood, respiratory, and sterile body fluids) were received for microbiological culture, excluding duplicate samples from the same patients, of which 668 (82%) samples were culture negative. Overall, 146 (17.9%) samples were culture positive. Out of these 146 patients, 54.1% acquired SBIs within the first week of hospitalization within an average of 4.1 days but after 48 hours of hospitalization, and 29 (19.9%) patients acquired infections after 7 days of hospitalization, so these (74%) could be classified as SBIs or hospital-acquired infections. However, 38 patients (26%) acquired infections within 48 hours of hospitalization and hence could be categorized as community-acquired infections or bacterial coinfections. The detailed profile of secondary infection pathogens (Table 3, Figure 1).

A total of 150 bacterial isolates were obtained from the 146 samples, out of which 7 were coagulase-negative staphylococcus (CoNS), which was excluded as a common contaminant due to its absence from repeat samples; thus, 143 significant organisms were obtained. Among the 143 bacterial isolates, the majority were gram-negative organisms i.e., 86%. *Acinetobacter species* was the commonest organism (51, 35.6%) (49 *Acinetobacter baumannii* and 2 *Acinetobacter nosocomialis*), followed by *Klebsiella pneumoniae* (26, 18.1%), *Pseudomonas species* (16,11%) (*Pseudomonas aeruginosa* [13]*, Pseudomonas stutzeri* [3]), *Escherichia coli* (9%) and *Burkholderia* species (4.2%) (*Burkholderia cepacia* [3], *Burkholderia* *cenocepacia* (2), *Burkholderia* *multivorans* [1]). Most of the *Acinetobacter species* (74.5%) were found in ICU patients (Table 4). Gram-positive organisms constituted 13.7% of the total 146 isolates, excluding CoNS.

### Antimicrobial resistance (AMR) profile of pathogens causing secondary infections

The AMR profiles of pathogens isolated from the clinical samples of COVID-19 patients are given in Table 5. The overall resistance rates among the gram-negative organisms ranged from 9% to 68.6% of all isolates. Among the gram-negative organisms, multidrug-resistant (MDR) isolates were present in 60.2% (74/123) of samples, and out of these, 51% (38/74) were admitted to ICU and 54% (40/74) succumbed to the disease. The isolation rates of carbapenem-resistant *Acinetobacter baumannii* (CRAB) and carbapenem-resistant *K. pneumoniae* (CRKP) were 65.4% and 64%, respectively. In all, 17.3% of *Acinetobacter*, 8% of *Klebsiella pneumoniae,* and 6.3% of *Pseudomonas* spp. were colistin resistant. Among the gram-positive organisms, methicillin-resistant *Staphylococcus aureus* was found in 5 out of 13 isolates. All *Staphylococcus aureus* isolates were susceptible to vancomycin and linezolid. Vancomycin-resistant *Enterococcus* was seen in 3 (37.5%) samples.

## Discussion

The emergence of the current COVID-19 pandemic has presented a formidable challenge to health systems and healthcare workers.^15,16^ Respiratory viral infections are known to predispose an individual to SBIs, leading to increased disease severity and mortality. Previous viral pandemics, such as the 1918 Spanish flu and the 2009 H1N1 influenza, were also associated with SBIs.^16^ Virus-mediated direct damage to the lung epithelium, an intense host immune response in the form of an aberrant cytokine storm, along with the subsequent use of steroids and immunomodulators, predispose these patients to developing SBIs. However, in our study, information regarding steroid status and administration of immunosuppressive agents in the patients was not available. The main cause of death in patients with COVID-19 is respiratory failure or multiple organ failure, and SBIs play a key role in this process.^17^

In the present study, the incidence and etiological profile of bacterial coinfections and secondary infections in patients with COVID-19 have been described. The rate of bacterial infections among total COVID-19 patients during the study period of 6 months was 7.9% which is similar to that reported in other studies.^18,19^ A recent Indian study conducted by Khurana et al. from northern India reported that 13% of COVID-19 patients had secondary infections.^18^ Similarly, a review by Rawson et al. showed a low incidence of coinfections in COVID-19 patients and that cytokine storm is an important factor in the deteriorating status of such patients.^5^ In a study conducted in Wuhan, among 1495 patients hospitalized with COVID-19, 102 (6.8%) patients had acquired SBIs, and almost half of them (49.0%) died during hospital stay.^19^ Similarly, in a retrospective study conducted by Hughes et al. in the UK, even though the incidence of bacterial coinfections in early COVID-19 hospital presentation was low as 3.2%, it increased to 6.1% throughout admission.^20^ Similarly, in our study, the rate of bacterial coinfections was 4.7% at the time of admission and increased to 13.3% after 48 hours of hospitalization. This highlights the fact that prolonged hospitalization is an important factor that makes the patients prone to developing SBIs, which also holds true for non-COVID patients. Moreover, this rate of hospital-acquired infections was similar in non-COVID areas during the same study period and also during pre-COVID times. This shows that prolonged hospitalization and suboptimal infection control practices could be the main culprits in causing these infections. It is a known fact that the greater the number of days of hospitalization, the greater the chances of getting hospital-acquired infections (HAIs).^20^ In this pandemic era, infection control practices are significantly affected due to high patient loads as well as lesser monitoring of these practices, both of which will lead to an increased rate of HAIs. Therefore, adequate infection control practices will play a major role in preventing these infections rather than initiating prophylactic antibiotics in all COVID-19 cases.

Regarding the organ systems affected by SBIs, the lungs were the major site involved in several studies.^18,21^ This may be related to the decreased airway defense function after a COVID-19 infection.^21^ A similar observation was reported in a study by Sharifipour et al. in Iran, where all 19 critical patients of confirmed COVID-19 infection were found to be positive for bacterial respiratory coinfections.^22^ In the Indian study by Khurana et al., positive respiratory cultures were seen in 83% of samples [18]. Secondary infections are more commonly reported in severely ill hospitalized COVID-19 patients, particularly in the ICU setting. A study conducted in Wuhan revealed secondary infections in 31% of ICU patients and 10% in the overall admitted patients.^23^ Similarly, the study by Li et al. from Wuhan concluded that 26.7% of COVID-19 patients in ICU settings were more likely to acquire SBIs as compared with patients in non-ICU settings.^19^ In our study, 25.2% of COVID-19 in ICU setting acquired bacterial infections which is in close agreement with previous studies. In terms of mortality, in previous studies, almost half of COVID-19 patients with SBIs died during hospitalization, which also agrees with our study (53.4%),^18,19^ and the cause of death in all such patients was found to be septic shock along with post-COVID ARDS. The mortality rate in COVID-19 patients without bacterial infections were 24% and this difference was statistically significant highlighting the fact that SBIs play a major causative role in patient mortality.

The etiological distribution in these patients was also a reflection of that seen in the non-COVID areas of the hospital as gram-negative organisms, especially *Acinetobacter baumannii* were most commonly isolated from the ICU settings of the non-COVID zone of the institute during the study period. Out of the total 143 isolates obtained during the study period, the majority (86%) were gram-negative organisms, as seen in other studies.^18,19,22^ The most common bacteria seen were *Acinetobacter baumannii*, *Klebsiella pneumoniae*, and *Pseudomonas species*, in agreement with a previous study conducted in Wuhan.^19^ Infection rates with gram-positive organisms were low in our study, as only 14% isolates were gram-positive. This was in close agreement with the study conducted by Sharifipour et al., in which only 10% of the total isolates were gram positive.^22^ The majority of the *Acinetobacter species* (74.5%) isolated in our study were found in ICU patients. *Acinetobacter* *baumannii* has been considered as one of the most common causes for HAIs mainly in ICU settings as it predominantly affects the debilitated patients in intensive care units.^24^ Moreover, the increased resistant strains of *Acinetobacter baumannii* poses a real threat to these patients.

It is always a dilemma whether to start prophylactic antibiotics in COVID-19 patients for superinfections or not. There are always institutional antimicrobial recommendations regarding the use of different types of antibiotics in different settings, but during COVID times, due to the fear of SBIs in COVID-19 patients, these recommendations are not being followed, and most of the COVID-19-positive patients are receiving empirical antibiotics. In the present study, approximately 75% patients had already received empirical antibiotics before the samples were sent for analysis. According to previously published studies, almost 72% of COVID-19 patients received empirical antimicrobial therapy, with no details of antimicrobial stewardship interventions.^5,10^ According to a recent living meta-analysis on bacterial coinfections in COVID-19 patients, in which 24 studies were included, the reported bacterial infection rate was 6.9%.^25^ This meta-analysis concluded that bacterial co-infection was not frequent in hospitalized COVID-19 patients and that the majority of them may not require empirical antibiotics.

Undue antibiotic prescription in viral infections leads to the problem of an increase in antimicrobial resistance. In our cohort, a high prevalence of drug-resistant isolates was seen. The majority (50.7%) of the pathogenic organisms reported were MDR nosocomial pathogens, in agreement with previous studies,^18,19,22^ and more than half of the patients infected (54%) succumbed to the disease. Sharifipour et al. in their study also emphasized the concern of MDR bacterial infections due to *A*. *baumannii,* especially in ICU patients.^22^ The isolation rates of CRAB and carbapenem-resistant *Klebsiella pneumoniae* (CRKP) in the present study were 56.9% and 32%, which is comparatively less than the previous study conducted in Wuhan (91.7% and 76.6%, respectively).^19^ When patients with COVID-19 suffer from SBIs, the chances of infections by drug-resistant strains are quite high due to the increased use of empirical antibiotics in these cases. This is because of very limited data available regarding the role of secondary bacterial infections in the severity of COVID-19.^5,10^ Since many studies, including ours, have shown the lesser prevalence of gram-positive organisms as a cause of SBI, restricted use of empirical broad-spectrum antibiotics for gram-positive organisms should be adhered to in our settings.

In our country, with an existing high baseline AMR burden, the COVID-19 pandemic has further intensified this issue due to many factors *viz*, the practice of sending inadequate microbiological cultures due to lesser invasive procedures being undertaken in these patients as a part of infection control measures and irrational use of empirical antibiotics among these patients. The increased threat of AMR poses a healthcare burden that ultimately leads to an economical loss for any nation. According to the Centers for Disease Control and Prevention (CDC), antimicrobial resistance adds a 20-billion-dollar surplus in direct healthcare costs in the United States, which is exclusive of approximately 35 billion dollars in loss of productivity annually.^26^ To reduce the prevalence of AMR, targeted and culture-based antibiotic therapy should be the usual practice. A retrospective study conducted by Evans et al. supported the use of narrow-spectrum antibiotics, which should be rapidly discontinued once the clinical picture and diagnostic work-up makes bacterial involvement unlikely.^27^ Moreover, in the given study, the rate and pattern of bacterial infections in COVID-19 patients was similar to those in non-COVID patients and comparable to those in pre-COVID times, so basic infection control practices to prevent healthcare associated infections along with culture-based initiation of antibiotics rather than irrational use of empirical antibiotics should be a norm in this COVID-19 era.

## Conclusion

To conclude, we see that the overall rate of bacterial infections in COVID-19 patients in our study was lower than that in previous studies. Therefore, judicious use of antimicrobials should be the norm in patients with respiratory viral infections. A rational approach would be to adhere to the practice of initiating culture-based guidance for antimicrobial therapy, to restrict unnecessary empirical antimicrobial therapy, and to adhere to strict infection control practices. Training of hospital staff regarding the above points should be performed regularly so that these can be implemented in patient care. Further, large-scale studies are needed regarding the status of bacterial infections in COVID-19 patients and to determine the role of empirical antibiotics in such patients.

## Declarations

### Conflicts of interest

The author(s) declared no potential conflicts of interest with respect to the research, authorship, and/or publication of this article.

### Source of funding

The author(s) received no financial support for the research, authorship, and/or publication of this article.

## Figures and Tables

**Table 1:** Demographic and outcome data of COVID-19 patients (n = 146) with bacterial coinfections and secondary infections during hospitalization for COVID-19.

**Characteristics**	**ICU (n = 94)**	**NON-ICU (n = 52)**

**Age**	48 ± 17.15	48 ± 17.48

**Sex**		

** Male**	58	39

** Female**	36	13

**Discharged**	45 (47.8%)	23 (44.2%)

**Death**	49 (52.1%)	29 (55.8%)


* Data is represented as mean ± standard deviation with percentage within ()

**Table 2:** Comparison of COVID-19 patients (n = 814) based on severity of illness and mortality

	**Patients with bacterial infections (n = 146)**	**Patients without bacterial infections (n = 668)**	**p-value**

**Death**	78 (53.4%)	160 (24%)	< 0.00001

**Discharged**	68 (46.6%)	508 (76%)	

**ICU admission**	94 (64.4%)	130 (19.5%)	< 0.00001


*Data are represented as number of patients with percentage within ().

# A *p-*value < 0.05 was considered statistically significant

**Table 3:** Etiological distribution of bacterial infections in patients hospitalized with COVID-19 (n = 146).

**Organism**	**Blood culture (n = 74)**	**Respiratory (n = 63)**	**Sterile Body fluids (n = 6)**	**Total no. of isolates (n = 143)**

***Acinetobacter species***	23(31.8%)	28 (44.4%)		51(35.7%)

***Klebsiella pneumoniae***	11(14.9%)	14 (22.2%)	1 (16.7%)	26 (18.2%)

***Pseudomonas species***	9 (12.2%)	7(11.1%)		16 (11.2%)

***Escherichia coli***	9 (12.2%)	2(3.2%)	2(33.3%)	13 (9.1%)

***Burkholderia species***	4(5.4%)	2 (3.2%)		6 (4.2%)

***Stenotrophomonas maltophilia***	1(1.4%)	4(6.3%)		5 (3.5%)

***Providencia rettgeri***	1(1.4%)	1(1.6%)		2 (1.4%)

***Morganella morganii***	1(1.4%)			1 (0.7%)

***Proteus mirabilis***	1(1.4%)			1 (0.7%)

***Pandoraea***	1(1.4%)			1(0.7%)

***Citrobacter koseri***		1 (1.6%)		1(0.7%)

***Staphylococcus aureus***	68.1%)	4 (6.3%)	3 (50%)	13 (9%)

***Enterococcus species***	7 (9.5%)			7 (4.9%)


* Data is represented as number of isolates with percentage within ().

**Table 4:** Area wise distribution of organisms (n = 143) causing bacterial coinfections and secondary infections.

Organism (n)	ICU (n=85)	Non-ICU (n=58)

***Acinetobacter species*** (51)	38 (44.7%)	13 (22.4%)

***Klebsiella pneumoniae*** (26)	15 (17.6%)	11 (19%)

***Pseudomonas species*** (16)	5 (5.9%)	11 (19%)

***Escherichia coli*** (13)	4(4.7%)	9 (15.5%)

***Burkholderia species*** (6)	2 (2.4%)	4 (6.9%)

***Stenotrophomonas maltophilia*** (5)	5 (5.9%)	0

***Providencia rettgeri*** (2)	2(2.4%)	0

***Morganella morganii*** (1)	0	1(1.7%)

***Proteus mirabilis*** (1)	0	1(1.7%)

***Pandoraea species*** (1)	0	1 (1.7%)

***Citrobacter koseri*** (1)	1(1.2%)	0

***Staphylococcus aureus*** (13)	8 (9.4%)	5 (8.6%)

***Enterococcus species*** (7)	5 (5.9%)	2 (3.4%)


* Data are represented as number of isolates with percentage within ().

**Table 5:** Antimicrobial resistance profile of gram-negative isolates (n = 117) causing bacterial infections in COVID-19 patients.

**Antimicrobials**	**Acinetobacter (n = 51)**	**Klebsiella pneumoniae (n = 26)**	**Pseudomonas species (n = 16)**	**Escherichia coli (n = 13)**	**Stenotrophomonas maltophilia (n = 5)**	**Burkholderia species (n = 6)**

**Major gram-negative organisms, N = no. of resistant isolates**						

**Amikacin**	18	10	4	3		

**Ceftazidime**	28	14	4	4	2	2

**Cefepime**	40	18	6	7		

**Piperacillin-tazobactam**	41	17	7	6		

**Imipenem**	40	16	8	6		

**Meropenem**	34	17	4	5	3	

**Cefoperazone-Sulbactam**	39	17	8	6		

**Colistin**	9	2	1	0		

**Ciprofloxacin**	39	18	6	9		

**Chloramphenicol**					1	2

**Cefotaxime**						1

**Levofloxacin**					0	0

**Minocycline**					0	0

**Cotrimoxazole**					0	1

